# The impact of the COVID-19 pandemic on wildlife–aircraft collisions at US airports

**DOI:** 10.1038/s41598-023-38451-9

**Published:** 2023-07-18

**Authors:** Levi Altringer, Sophie C. McKee, Jason D. Kougher, Michael J. Begier, Stephanie A. Shwiff

**Affiliations:** 1 Animal and Plant Health Inspection Service, Wildlife Services, United States Department of Agriculture, National Wildlife Research Center, 4101 LaPorte Avenue, Fort Collins, CO 80521 USA; 2 Department of Economics, Colorado State University, Fort Collins, CO 80523 USA; 3 United States Department of Agriculture, Animal and Plant Health Inspection Service, Wildlife Services, Airport Wildlife Hazards Program, Washington, DC 20250 USA; 4 United States Department of Agriculture, Animal and Plant Health Inspection Service, Wildlife Services, Airport Wildlife Hazards Program, 6100 Columbus Avenue, Sandusky, OH 44870 USA

**Keywords:** Natural hazards, Animal behaviour

## Abstract

Exploiting unprecedented reductions in aircraft movements caused by the COVID-19 pandemic, we investigated the relationship between air traffic volume and the frequency of wildlife-aircraft collisions, or wildlife strikes, at the 50 largest airports in the United States. During the COVID-19 months of 2020 (March–December), both air traffic volume and the absolute number of wildlife strikes were reduced. The net effect of these two movements, however, was an increase in the wildlife strike rate from May 2020–September 2020. This increase was found to be most pronounced at airports with larger relative declines in air traffic volume. We concluded that the observed increase in the wildlife strike rate was, at least in part, generated by risk-enhancing changes in wildlife abundance and behavior within the airport environment. That is, wildlife became more abundant and active at airports in response to declines in air traffic volume.

## Introduction

Wildlife-aircraft collisions, commonly referred to as wildlife strikes or bird strikes, are relatively rare events that pose considerable safety and economic risks within the aviation industry. Damaging wildlife strike events generate substantial repair costs as well as downtime for aircraft and commercial aircraft passengers^1–3^. In the most severe and rare instances, wildlife strikes can cause injury and even loss of life^4^. Given the potentially dramatic consequences, much work has been done to reduce the likelihood of wildlife strikes at airports—e.g., identifying risk factors and best practices for mitigating wildlife hazards^5–9^. What is less understood, however, is how air traffic volume itself might affect wildlife strike frequency via changes in the abundance and behavior of wildlife within the airport environment.

The number of wildlife strikes occurring at a given airport is a function of air traffic volume, as well as the use of the airport environment by wildlife—e.g., abundance and species composition^10^. The influence of these two factors, separately, is intuitive. Holding the abundance and behavior of wildlife constant, one would expect to observe an increase in the absolute number of wildlife strikes given an increase in air traffic volume. Alternatively, an increase in the abundance of wildlife on or near an airfield, holding air traffic volume constant, would also increase the absolute number of wildlife strikes. What is less clear, however, is the synergistic relationship between air traffic volume and the abundance and behavior of wildlife. For instance, if aircraft movements themselves deter wildlife from the airport environment, then decreases in air traffic volume could simultaneously increase the abundance of wildlife on or near the airfield. It is plausible, then, that a decrease in air traffic volume might have little to no effect on the absolute number of wildlife strikes due to increases in wildlife abundance within the airport environment, in which case the wildlife strike rate—i.e., the number of wildlife strikes per aircraft movement—would actually increase. In other words, the relationship between air traffic volume and the number of collisions with wildlife might be non-linear^11,12^.

**Figure 1:**
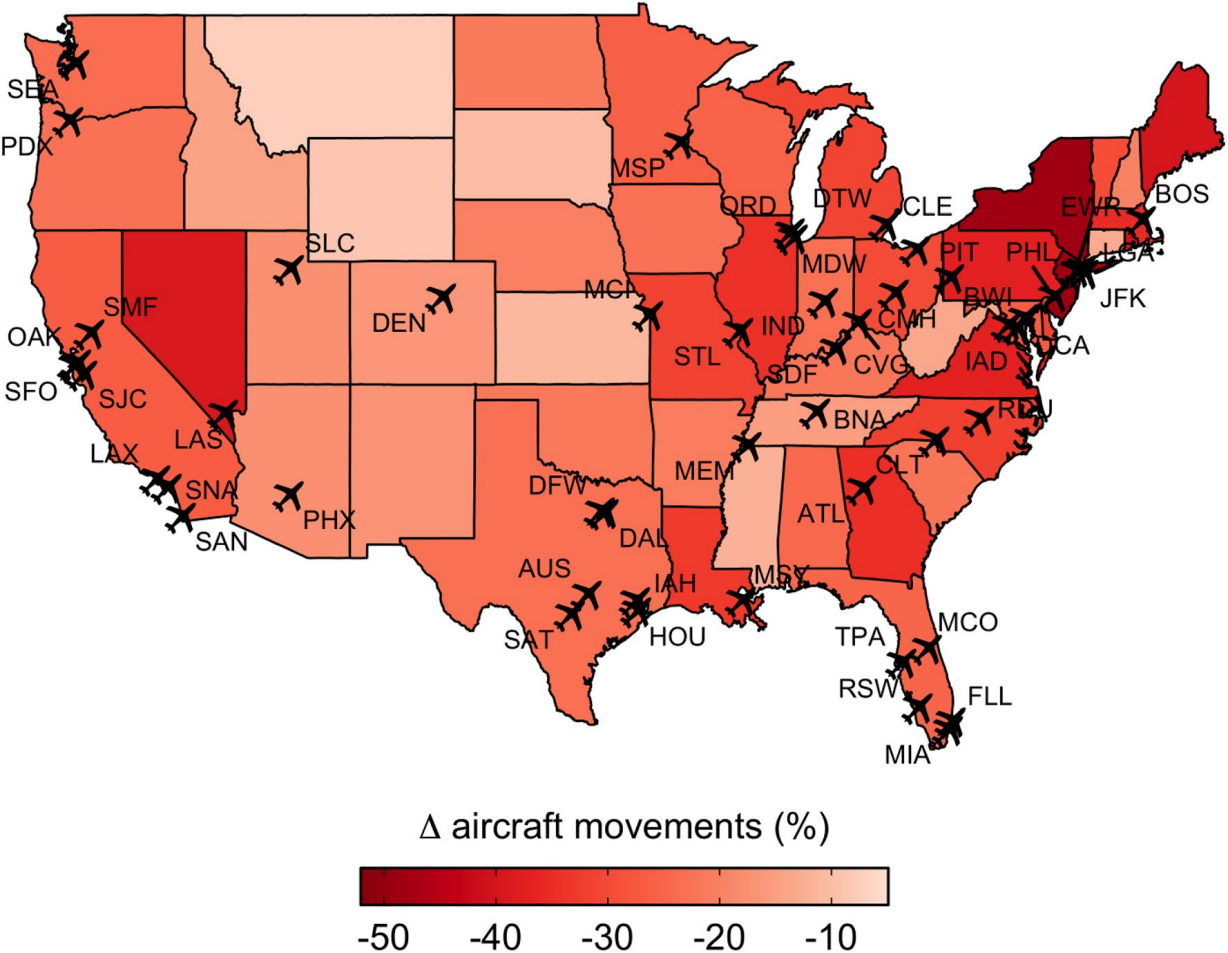
Spatial distribution of sample airports and state-level effects of the COVID-19 pandemic on air traffic volume across the contiguous United States. Percent change (Δ) in aircraft movements (total number of takeoffs and landings) is measured between the pre-pandemic period March 2019–December 2019 and the pandemic period March 2020–December 2020. All states experienced declines in air traffic volume beginning in March 2020. The largest changes in air traffic volume were observed in the densely populated District of Columbia (− 66.7%), New Jersey (− 48.4%), and New York (− 48.0%) while the smallest changes were observed in some of the least densely populated states such as Montana (− 7.4%), Wyoming (− 8.7%), and South Dakota (− 10.1%).

Up until now, an investigation into the relationship between air traffic volume and wildlife strike frequency would have had to rely on cross-airport comparisons, which are likely to be biased by location-specific factors that generate differences in the frequency of wildlife strikes and are correlated with differences in air traffic volume—i.e., omitted variable bias. For example, airports with lower air traffic volume are also more likely to be located in relatively wildlife-rich suburban or rural areas^5,9^. The COVID-19 pandemic, however, presents a potentially useful natural experiment^13^. The early stages of the COVID-19 pandemic produced unprecedented reductions in modern human mobility^14^. Not least affected by the pandemic-induced “anthropause”^15^ were large commercial airports which experienced unprecedented declines in air traffic volume over the COVID-19 months of 2020—i.e., March 2020 to December 2020—and beyond^16^ (Fig. 1). Thus, instead of relying on problematic cross-airport comparisons, the pandemic-induced reduction in air traffic volume that began in March 2020 allows an investigation into the relationship between air traffic volume and wildlife strike rates using within-airport comparisons that hold location-specific confounding factors constant.

Recent research suggests that wildlife was quick to respond to reductions in human activity over the course of the pandemic, particularly during the early lockdown stages^12,17–20^. Of particular interest to our investigation, is the finding that birds became more abundant in urban areas, near major roads, and, importantly, near airports^19^. It has yet to be determined, however, if reductions in air traffic volume and the increased abundance of wildlife near, or within, the airport environment had a measurable impact on the rate at which wildlife-aircraft collisions occurred. We speculated that, in response to the dramatic decline in air traffic volume over the COVID-19 months of 2020 (Figs. S1, S2), the count of wildlife strikes was likely to fall. Given the potential effects of air traffic volume on wildlife abundance and behavior, however, we hypothesized that the net effect of reduced air traffic volume and declines in the count of wildlife strikes would be an increase in the wildlife strike rate.

There are several plausible hypotheses as to why the net effect of reduced air traffic volume might be an increase in the wildlife strike rate. Air traffic volume and the associated operational activities at airports likely act as a form of harassment—i.e., deterrent—for wildlife within the airport environment^11,20^. Decreases in air traffic volume would reduce these stressors, making the airport environment more appealing to wildlife and, therefore, result in collision-enhancing changes to wildlife abundance and behavior. For birds within the airport environment, stressors such as chemical and noise pollution might also affect nesting decisions and reproductive success^17,21,22^, and reductions in air traffic volume might make near and on-airfield nesting more attractive. This potential mechanism is particularly relevant to our investigation since the most pronounced reductions in air traffic volume were coincident with the nesting and fledging of birds in most of the study area—i.e., March–July in the contiguous United States (Figs. S1, S2). Apart from behavioral responses from wildlife, pandemic-related changes in reporting behavior and management activities are additional plausible explanations, though there exists no data or accounts to supply insight in this direction. We explore these alternative explanations further in our discussion.

Utilizing the unprecedented reductions in aircraft movements caused by the COVID-19 pandemic, our study investigated the relationship between air traffic volume and the frequency of wildlife-aircraft collisions vis-à-vis changes in the wildlife strike rate at the 50 largest airports in the United States (Fig. 1). Ours is not the first study to exploit pandemic-induced human inactivity to show that, all else equal, traffic volume may be nonlinearly associated with the frequency of wildlife collisions. For example, and under a similar conceptual framework, state-level insurance data was used to show that wildlife-vehicle collision rates were elevated during the early stages of the COVID-19 pandemic in response to declines in motor vehicle traffic^12^. In our study, we extended this intuition to the realm of wildlife-aircraft collisions. We began with sample-wide and airport-specific comparisons of air traffic volume^23^, wildlife strikes^24^, and wildlife strike rates in the pre-pandemic—March 2019 to December 2019— and pandemic—March 2020 to December 2020—periods. Moving from the raw data, we then employed fixed-effect negative binomial regression models with panel data^25^, which controlled for pre-existing trends in wildlife strike rates, held constant location-specific confounding factors, and, therefore, provided a more precise estimate of directional changes in the wildlife strike rate during the COIVD-19 months of 2020. As such, our study provides novel insight into the relationship between air traffic volume and wildlife-aircraft collisions, offering important implications for the management of wildlife hazards at airports. From an ecological perspective, this research contributes to a growing body of literature that emphasizes and demonstrates the impact of modern human activity on wildlife and its behavior, with a particular interest in human-wildlife interactions over the COVID-19 pandemic of 2020^12–15,17–20,22^.

## Results

**Figure 2:**
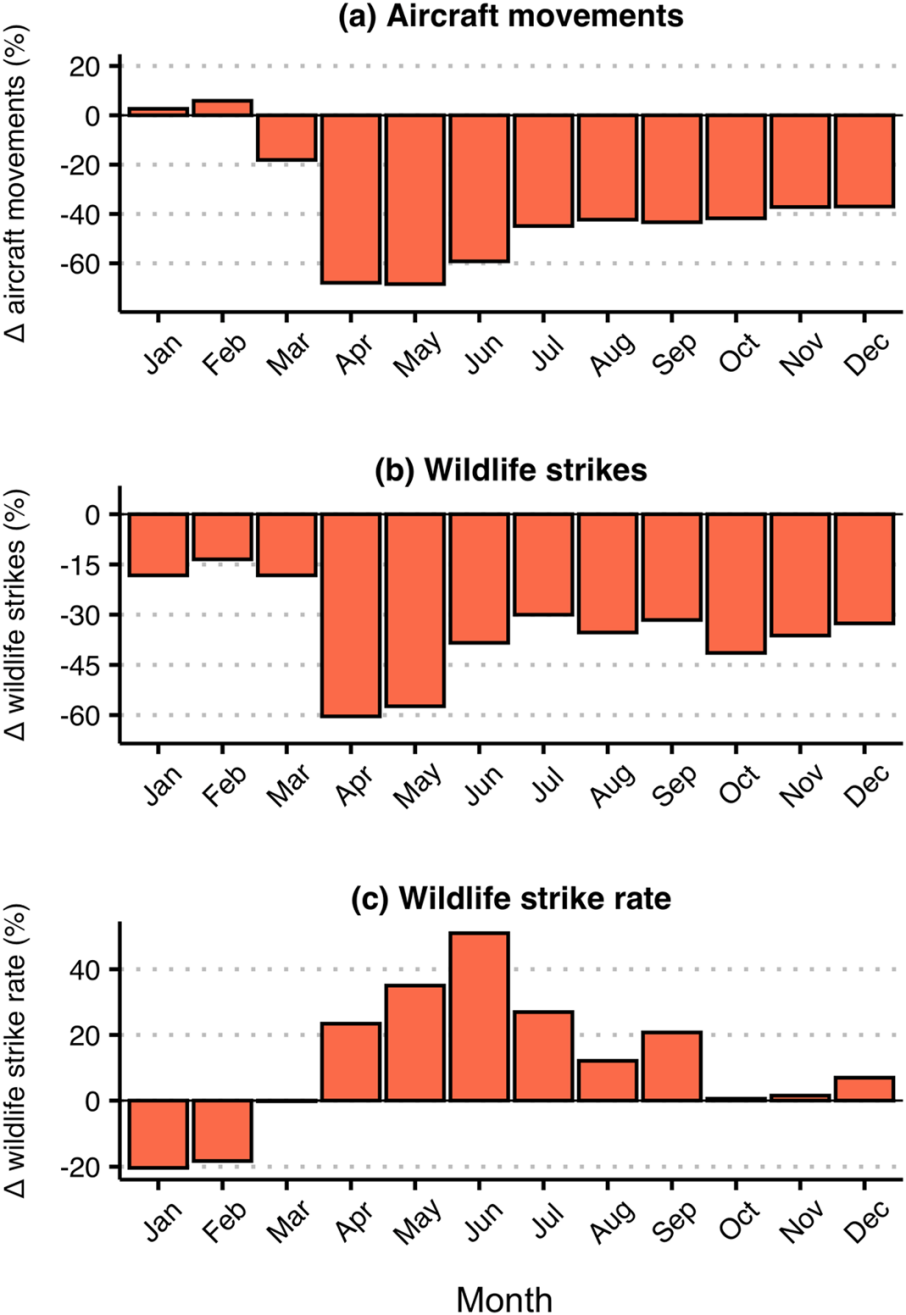
Month-specific effects of the pandemic among sample airports. Data is aggregated across all sample airports in this analysis. Percent changes (Δ) in (**a**) aircraft movements (total number of takeoffs and landings), (**b**) wildlife strikes (number of wildlife strikes reported to the NWSD), and (**c**) the wildlife strike rate (number of reported wildlife strikes per 100,000 movements) are measured between 2019 and 2020 for all months January–December.

To begin our investigation into the relationship between air traffic volume and wildlife strike frequency vis-à-vis the COVID-19 pandemic, we compared air traffic volume, wildlife strikes, and the wildlife strike rate in the pre-pandemic period—March 2019 to December 2019—to those observed during the COVID-19 pandemic—March 2020 to December 2020. We started by examining the sample as a whole and then turned to airport-level comparisons. A dramatic reduction in air traffic volume began in March of 2020 (Fig. 2a), the same month that the World Health Organization declared COVID-19 a pandemic and the Trump administration declared a national emergency. Among the airports in our sample, total air traffic volume quickly plunged to its pandemic low in April and May of 2020, when aircraft movements fell 67.9 and 68.4 percent, respectively, relative to April and May of 2019. By December of 2020, air traffic volume was still reduced by 37.0 percent. In response to the reduction in aircraft movements, the count of wildlife strikes also fell in 2020 relative to 2019 (Fig. 2b). Among the airports in our sample, wildlife strike counts were reduced by 60.4 and 57.4% in April and May of 2020, respectively. Between June and December of 2020, the absolute number of wildlife strikes were down 30 to 40% relative to 2019. While the absolute number of wildlife strikes fell, the net effect of the reduction in air traffic volume and the number wildlife strikes resulted in an increase in the wildlife strike rate (Fig. 2c). The overall sample increase in the wildlife strike rate lasted from April 2020–September 2020 and it was particularly pronounced in June 2020—49.8 percent increase—following the large reductions in air traffic volume that occurred in April 2020 and May 2020.

**Figure 3:**
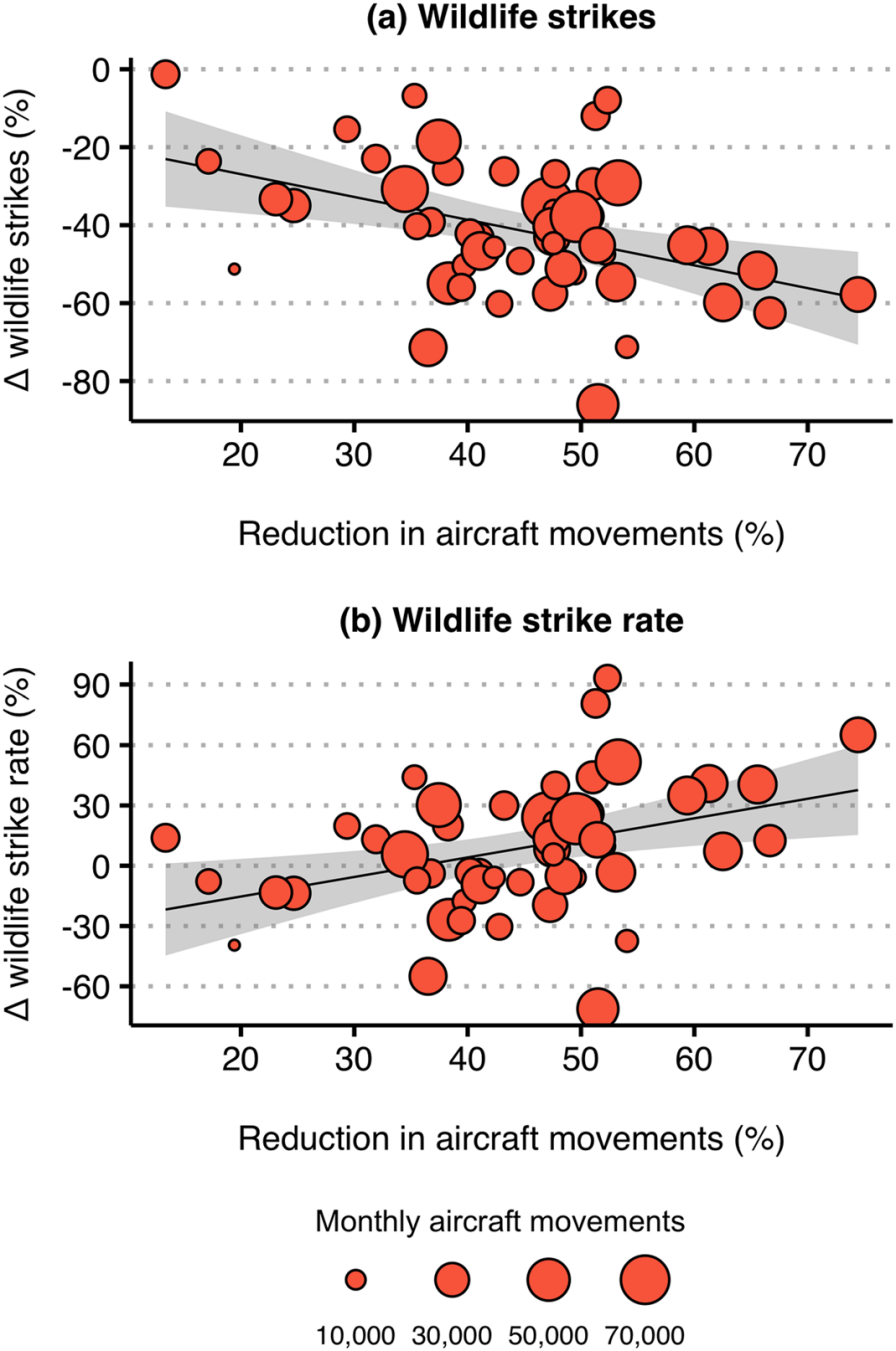
Bivariate relationship between reductions in air traffic volume and changes in wildlife strikes. Each point represents a sample airport, with the size of each point indicating operational size in 2019 as measured by the average monthly number of takeoffs and landings. The percent reduction in aircraft movements (number of takeoffs and landings) at each airport is measured between periods March 2019–December 2019 and March 2020–December 2020. Percent changes (Δ) in (**a**) wildlife strikes (number of wildlife strikes reported to the NWSD) and (%) the wildlife strike rate (number of reported wildlife strikes per 100,000 movements) at each airport are similarly measured between March 2019–December 2019 and March 2020–December 2020. Response curves are bivariate linear regression fits of the data, where the shaded area surrounding each curve is the associated 95% interval of confidence. A Pearson correlation test indicates that reductions in aircraft movements are significantly negatively correlated with changes in the absolute number of wildlife strikes (*r* =  − 0.420, *p* = 0.002). A similar test shows that reductions in aircraft movements are significantly positively correlated with changes in wildlife strike rates (*r* = 0.380, *p* = 0.006).

Disaggregating the sample, we then compared changes in air traffic volume, wildlife strikes, and the wildlife strike rate at the airport-level. Again, changes in air traffic volume, wildlife strikes, and the wildlife strike rate were determined by comparing measures in the pre-pandemic period—March 2019 to December 2019—to those observed during the COVID-19 pandemic—March 2020 to December 2020. We found that both air traffic volume and the absolute number of wildlife-aircraft collisions were reduced across all sample airports during the COVID-19 months of 2020 (March–December), while only 28 airports experienced an increase in the wildlife strike rate over the same period (Fig. 3). The airports that experienced the largest reductions in air traffic volume were also those that, on average, experienced the largest reductions in the absolute number of wildlife strikes (*r* =  − 0.420, *p* = 0.002) (Fig. 3a). Oppositely, the airports that experienced the largest reductions in air traffic volume were also those that, on average, experienced more positive changes in the wildlife strike rate (*r* = 0.380, *p* = 0.006) relative to 2019 (Fig. 3b).

**Figure 4:**
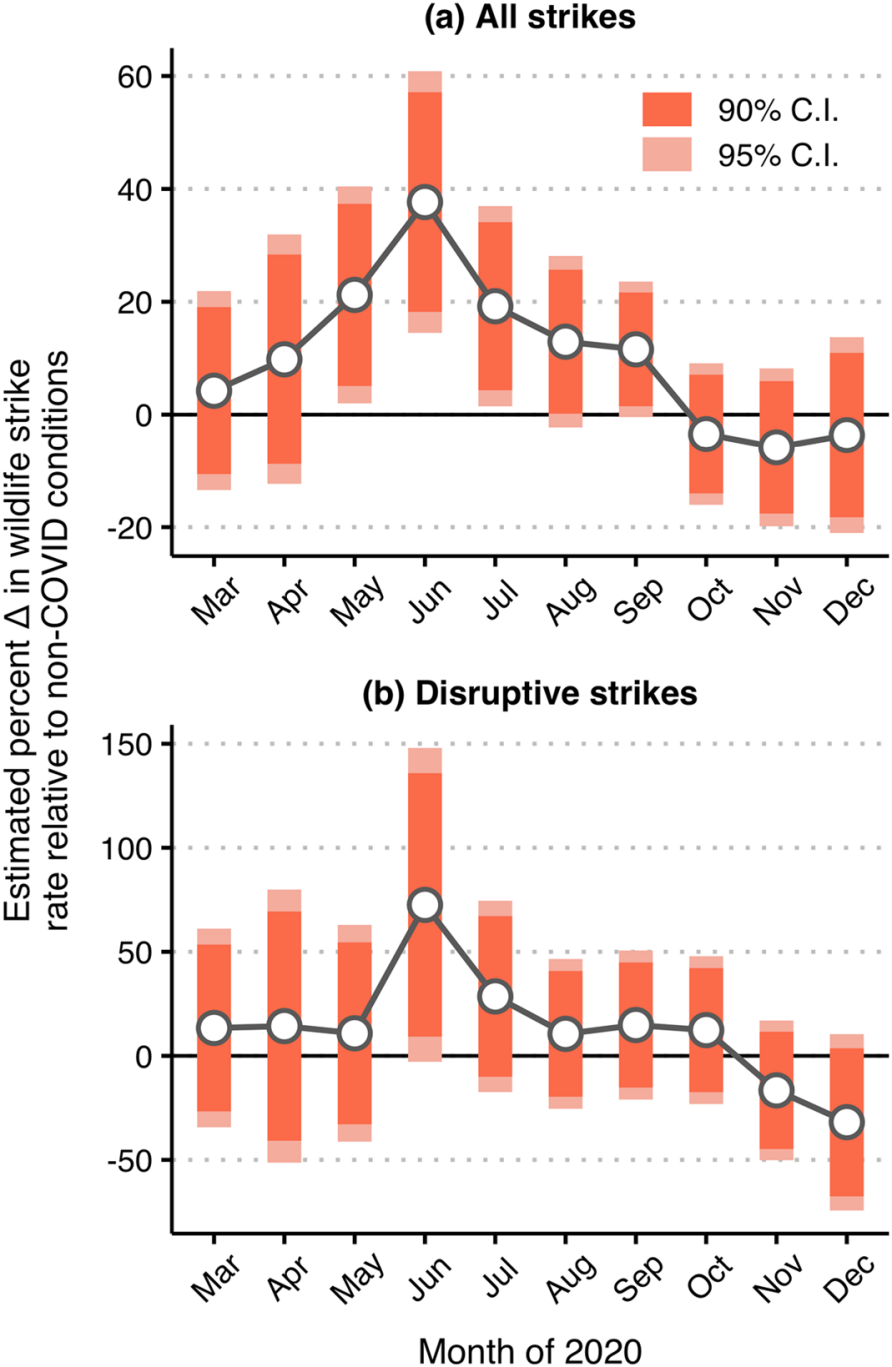
Month-specific model estimated changes in the (**a**) overall and (**b**) disruptive wildlife strike rate during the COVID-19 months of 2020. The connected points in panels (**a,b**) report the model estimated percent change in the wildlife strike rate over the COVID months of 2020, all else equal. The error bars provide the associated 90% and 95% intervals of confidence that surround each estimate. These estimates are derived from the incident rate ratios (IRRs) generated via fixed-effect negative binomial regression models (Tables S3, S4). Specifically, they are calculated [IRR − 1] × 100 to supply a direct interpretation.

Moving from the raw data, the estimates produced by our first set of negative binomial regression models suggested that, on average, the overall wildlife strike rate was significantly elevated across airports in our sample from May 2020 to September 2020 (Fig. 4a). Our preferred model included airport fixed-effects to account for unobserved location-specific heterogeneity, region-by-month fixed effects to control for region-specific seasonality, and a general time trend to adjust for pre-existing trends (Tables S2a, S3). The largest estimated increase was observed in June, where the wildlife strike rate was estimated to be 38 percent higher relative to non-COVID conditions, 90% CI [18.2, 57.1] (Table S3). This considerable increase in the wildlife strike rate comes directly after April 2020 and May 2020, when reductions in air traffic volume were most severe (Fig. 2, Fig. S2). While the overall wildlife strike rate increased during May 2020–September 2020, there was no significant evidence of a similarly dramatic increase in the disruptive wildlife strike rate (Fig. 4b). For the disruptive wildlife strike rate, our preferred model included airport fixed effects to account for unobserved location-specific heterogeneity and general month fixed effects to control for seasonality (Tables S2b, S4). Only in June 2020, just after the severe reductions in air traffic volume during April 2020 and May 2020, did we observe an increase—72%, 90% CI [9.2, 135.9]—in the disruptive wildlife strike rate relative to non-COVID conditions (Table S4). Again, disruptive wildlife strikes are those that are characterized by one or more of the following: repair costs, non-repair (other) costs, damage, a negative effect on the flight, or aircraft downtime.

**Figure 5:**
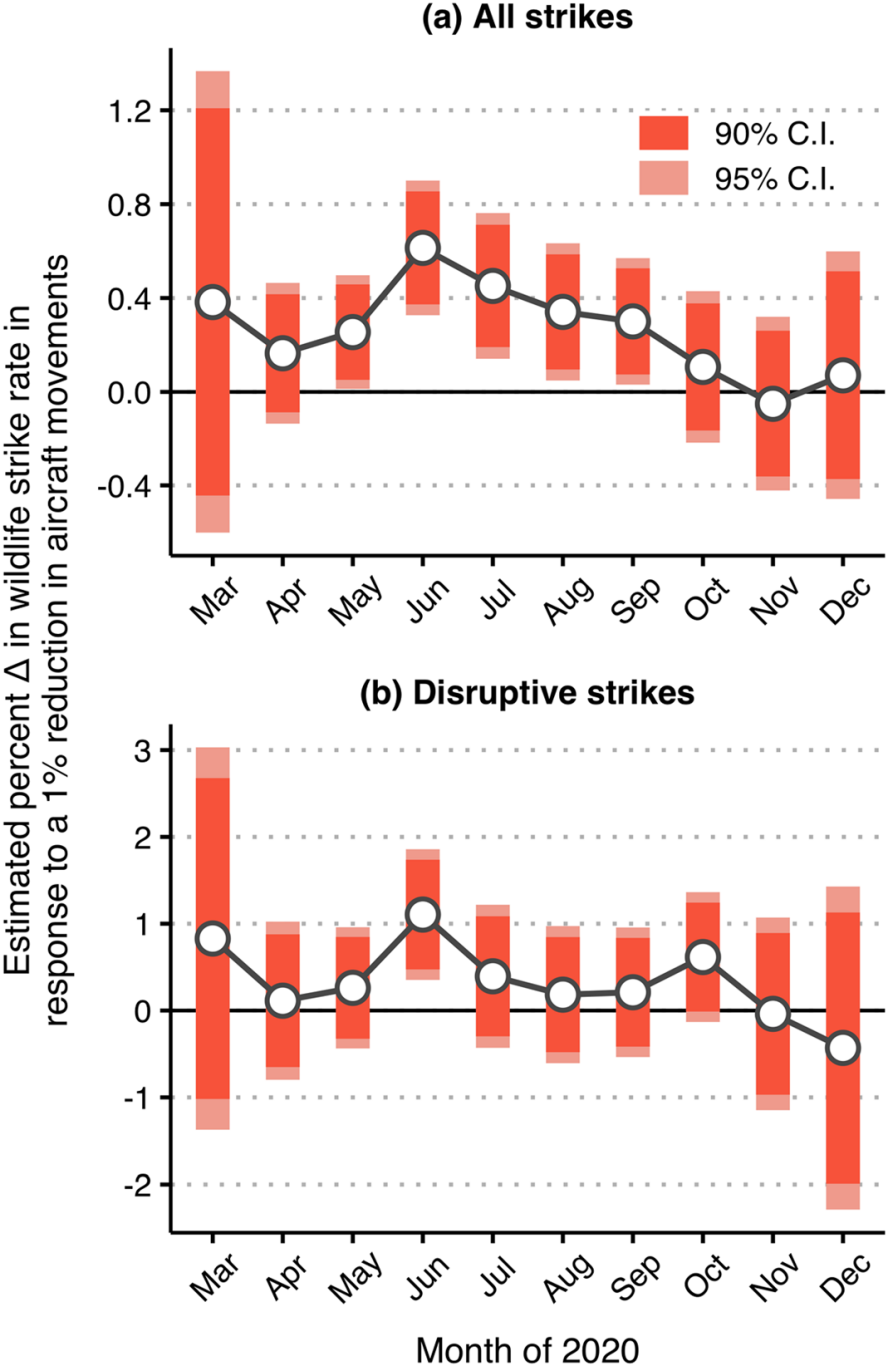
Month-specific model estimated relationship between reductions in air traffic volume and the (**a**) overall and (**b**) disruptive wildlife strike rate during the COVID-19 months of 2020. The connected points in panels (**a,b**) report the model estimated percent change in the wildlife strike rate that is associated with a 1% reduction in aircraft movements, all else equal. The error bars provide the associated 90% and 95% intervals of confidence that surround each estimate. These estimates are derived from the incident rate ratios (IRRs) generated via fixed-effect negative binomial regression models (Tables S6, S7). Specifically, they are calculated: [IRR − 1] × 100.

Finally, to empirically link the observed increases in the wildlife strike rate to pandemic-induced changes air traffic volume, our second set of negative binomial regression models investigated month-specific relationships between reductions in air traffic volume and deviations in the overall and disruptive wildlife strike rates (Fig. 5). From our model selection exercise, our preferred model for the overall wildlife strike rate included airport fixed effects, region-by-month fixed effects, and a general time trend as controls (Tables S5a, S6). For the disruptive wildlife strike rate, our preferred model included airport fixed effects and general month fixed effects as controls (Tables S5b, S7). We found that, on average, reductions in air traffic volume were significantly positively associated with deviations in the overall wildlife strike rate, all else equal, during May 2020–September of 2020 (Fig. 5a). Most notably, a 1% reduction in air traffic volume was associated with an average 0.61% increase in the wildlife strike rate across the airports in our sample during June 2020, 90% CI [0.37, 0.85] (Table S6). For reference, the average reduction in air traffic volume during June of 2020 was approximately 57%. By October 2020–December 2020, however, deviations in the overall wildlife strike rate were statistically unrelated to reductions in air traffic volume. Further, and similar to previously discussed results, reductions in air traffic volume were largely statistically unrelated to changes in the disruptive wildlife strike rate, all else equal (Fig. 5b), with the exception of June 2020, when a 1% reduction in air traffic volume was associated with an average 1.1% increase in the disruptive wildlife strike rate, 90% CI [0.5, 1.7] (Table S7).

## Discussion

Taken as a whole, the airports in our sample experienced (1) a decrease in air traffic volume, (2) a decrease in the absolute number of wildlife-aircraft collisions, and (3) an increase in the wildlife strike rate during the COVID-19 months of 2020 relative to the previous year (Fig. 2). Similar to other studies of wildlife-vehicle collisions during the COVID-19 pandemic^12^, we observed a severe decline in traffic volume during April and May 2020 that was followed by a substantial increase in wildlife collision rates during May 2020–September 2020. Moving from the sample-wide analysis, we made use of the fact that pandemic-induced reductions in air traffic volume were more pronounced at some airports relative to others (Figs. S1, S2). We hypothesized that the count of wildlife strikes would be reduced at those airports that experienced the largest reductions in air traffic volume over the COVID-19 pandemic. On the other hand, given the hypothesized effects of air traffic volume on wildlife abundance and behavior, we anticipated that the same airports that experienced the largest reductions in air traffic volume would also have experienced increases in wildlife strike likelihood, as measured through the wildlife strike rate. We found support for both of these hypotheses among the airports in our sample, with reductions in air traffic volume being significantly negatively correlated with changes in wildlife strike counts and significantly positively correlated with changes in wildlife strike rates across airports (Fig. 3).

The fact that not all airports experienced increases in the wildlife strike rate, despite all experiencing a decrease in air traffic volume, was not entirely contradictory with the hypothesized impact of air traffic volume on wildlife-aircraft collisions vis-à-vis risk-enhancing changes in wildlife abundance and behavior. First, given the heterogeneous impact of the pandemic on airport operations, some airports may not have had large enough reductions in traffic volume to initiate a response from wildlife. Second, and in relation the previous point, aggregating airport-level data across the COVID-19 months of 2020 (March–December) is likely to mask month-specific effects when reductions air traffic volume were more ubiquitous across sample airports—i.e., April to June 2020 (Fig. S2). Third, simple comparisons of pre-pandemic (2019) and pandemic (2020) data do not account for pre-existing trends across airports that confound the observed impact of the COVID-19 pandemic via simple year-over-year comparisons.

The fixed-effect negative binomial regression models allowed us to address issues of omitted variable bias, control for pre-existing trends, account for region-specific phenology, and discern the month-specific impact of the pandemic on the wildlife strike rate via estimated average effects across airports. On average, and all else equal, the overall wildlife strike rate was significantly elevated during May 2020–September 2020, with the largest estimated effect being observed in June 2020 (Fig. 4a). Further, deviations in the overall wildlife strike rate were significantly positively associated with reductions in aircraft movements during the COVID-19 months May 2020–September 2020 (Fig. 5a). The months May–September are the months that, even prior to the pandemic, are relatively “bird-rich” from a wildlife strike risk perspective (see Table S1). Thus, it was no surprise that the hypothesized impact of pandemic-induced reductions in air traffic volume dissolved in the late fall and winter months, when wildlife is generally less abundant and active.

Relative to the overall wildlife strike rate, the disruptive wildlife strike rate did not systematically deviate from pre-COVID expectation during the COVID-19 months of 2020, with the exception of June 2020 (Figs. 4b, 5b). There are two plausible explanations for the estimated stability of the disruptive strike rate over the COVID-19 months of 2020. First, from an ecological perspective, reductions in air traffic volume might not affect the abundance and behavior of all species equally. In other words, wildlife that pose a lesser hazard—i.e., smaller in size or a lower propensity to flock^5,6,8,26^—might also be those which are more responsive to changes in near and on-airfield human activity. Second, and from a pure data perspective, disruptive wildlife strike events are much rarer and, in relative terms, more variable compared to the set of all wildlife strikes (Table S1). The rarity and variability of these more severe events may limit the ability of our models to precisely estimate any significant changes in the disruptive wildlife strike rate during the COVID-19 months of 2020. Relatedly, a supplementary size-specific analysis suggested that changes in the small- and medium-sized wildlife strike rate were the driver of our main results (Fig. S3). Among the airports in our sample, the small-sized wildlife (≤ 442 g) strike rate positively was significantly elevated, relative to non-COVID conditions, during May 2020–September 2020 while the medium-sized wildlife (≥ 443 g and ≤ 1500 g) strike rate was significantly elevated in June 2020 and July 2020. Given the known relationship between wildlife size and the likelihood of aircraft damage^5,6,8^, the estimated 115% average increase in the medium-sized wildlife strike rate during June 2020 (Fig. S3b) is likely the reason for the estimated increase in the disruptive strike rate during the same month.

The aptness of our models to properly identify deviations in wildlife strike rates during the COVID-19 months of 2020 relied on their ability to sufficiently predict variation in pre-COVID wildlife strike rates. One way to test the integrity of our models in this direction was to re-estimate each of our preferred models, but under the assumption that 2019 was the year affected by the COVID-19 pandemic—while excluding data from 2020. If our models sufficiently predict variation in pre- or non-COVID wildlife strike rates, we should not observe significant effects in “non-COVID” data. These tests are presented in the Supplementary Information, Figs. S4 and S5. From the exercise we concluded that our models are sufficiently robust in estimating pandemic-induced deviations in wildlife strike rates, particularly for the overall wildlife strike rate.

We posit that the observed increase in the wildlife-aircraft collision rate over the COVID-19 months of 2020 was, at least partially, generated by risk-enhancing changes in the abundance and behavior wildlife within the airport environment. This is entirely plausible given the recent finding that several species of birds were found in closer proximity to urban areas, airports in particular, during the early stages (March 2020–May 2020) of the COVID-19 pandemic^19^. The seasonal timing of the most extreme increases in the wildlife strike rate (May 2020–July 2020) was coincident with nesting and fledging of birds in the majority of the study area—i.e., contiguous United States. This invites conjecture that the settlement and nesting decisions made by birds in the early stages (March 2020–April 2020) of the COVID-19 pandemic may have contributed to the increase in wildlife-aircraft collisions in later months. Limited evidence suggests that this settlement and nesting hypothesis is not entirely unfounded^17^. Thus, the dispersal of young following near and on-airfield nesting activities is a potential reason for the strongest deviations in overall wildlife strike rate observed in May 2020–July 2020. Additionally, some have hypothesized that COVID-induced human inactivity was likely to have an effect on birds’ reproductive success via reductions in known stressors, including chemical, noise, and light pollution^22^. However, concerning the impact of the COVID-induced “anthropause” on birds’ reproductive success, the scant evidence is mixed^17,22^.

There are two alternative explanations for the observed increase in the wildlife strike rate during the pandemic, rather than hypothesized collision-enhancing changes in the abundance and behavior of wildlife within airport environments. The first alternative explanation concerns the detection, or reporting, of wildlife strikes^27,28^. Given the dramatic reduction in airport operations and air traffic volume, it is plausible that airline and airport personnel had more time to thoroughly inspect aircraft and runways which would increase the likelihood of detecting the occurrence of a wildlife-aircraft collision. The implications of this potential explanation are that (1) reporting is limited by time constraints placed on airline and airport personnel and (2) wildlife strikes occur more frequently than what can be determined from existing data. The second alternative explanation is that airports, in response to reduced air traffic volume, may have reduced wildlife hazard management activities which, in turn, could lead to a more prolific nesting season and a more inviting habitat for wildlife within the airport environment. If true, at least in part, then a portion of the observed increase in the wildlife strike rate over the COVID-19 months of 2020 are an indication of the effectiveness of wildlife hazard management at airports in reducing wildlife strike risk. A lack of accounts and data prohibit our ability to discern which explanation best accounts for the observed increase in the wildlife strike rate.

Given that our results corroborate those of previous studies^12,19^, we conclude that air traffic volume itself is likely to be important factor in determining the abundance and behavior of wildlife within the airport environment and, therefore, is responsible for a portion of the observed increase in wildlife strike frequency over the COVID-19 months of 2020. We posit that our study and its results hold considerable relevance from both an ecological and management perspective. This research contributes to a growing body of literature that emphasizes and demonstrates the impact of modern human activity on wildlife and its behavior, with a particular interest in human-wildlife interactions over the COVID-19 pandemic of 2020^12–15,17–20,22^. Intuitively, reductions in air traffic volume reduce the number of wildlife-aircraft collisions and offer a reprieve for wildlife in absolute terms. However, conceivable alterations in wildlife abundance and behavior^11,12,19^ can influence the rate of such incidents—specifically, the likelihood of a wildlife strike occurring during any particular flight. This latter point, that reductions in air traffic volume are associated with an increased likelihood of a wildlife strike at the flight-level, supplies an important implication from a management perspective. An extension of the logic suggests that airports with relatively low numbers of aircraft movements—e.g., small regional or general aviation airports—may face a greater relative risk simply due to lower volumes of air traffic, in addition to other important risk factors. Thus, while smaller airports may be less exposed from an overall risk perspective—i.e., lower traffic volume, less passengers per flight, and less expensive aircraft—wildlife managers should take note of how lower levels of air traffic volume might influence wildlife abundance and behavior, presenting particular challenges in mitigating wildlife hazards at their airports.

## Methods

### Data sources

To investigate the relationship between air traffic volume and wildlife strike frequency vis-à-vis pandemic-induced reductions in aircraft movements, we employed monthly air traffic volume and wildlife strike panel data for the 50 largest airports in the United States (Fig. 1) over the period January 2014–December 2020. The airports in our sample (1) account for roughly 35% of air traffic volume and 48% of wildlife strikes within the contiguous United States over the sample period, (2) provide representation in each of the North American flyways, and (3) ensure relatively reliable wildlife strike reporting^29^. Air traffic volume data, measured as the total number of aircraft movements—i.e., takeoffs and landings—were collected from the Air Traffic Activity Data System (ATADS)^23^. Wildlife strike data were collected from the National Wildlife Strike Database (NWSD), a database that has been collecting reported wildlife strikes since 1990 and currently contains over 260,000 strike records with information concerning the date, location, aircraft involved, and wildlife struck, among other details^24^.

The average airport in our sample, during the pre-pandemic period, experienced approximately 26,800 aircraft movements per month (Table S1) and there exists greater variation in air traffic volume between airports than within (SD^Overall^ = 15,849.4; SD^Between^ = 15,817.6; SD^Within^ = 2437.7). On average, air traffic volume was slightly elevated during the summer months—June, July, and August—and reached its yearly low in January and February. Wildlife strike counts exhibited a more dramatic seasonal pattern, with an overall average of 11.4 per month in the pre-pandemic period (Table S1). Specifically, the average count of wildlife strikes was lowest in January—roughly 4 wildlife strikes per month—and highest in August—18 per month. The sizeable seasonal fluctuation in wildlife strike counts generates a within-airport variation that is larger than between-airport variation (SD^Overall^ = 12.0; SD^Between^ = 6.9; SD^Within^ = 9.9). This underlines the importance of seasonal factors in explaining wildlife strike frequency. Combining wildlife strike counts and air traffic volume data, the average airport in our sample had a reported wildlife strike rate of 51.22 per 100,000 aircraft movements (Table S1). Among sample airports, the likelihood of a wildlife strike was largest in August with an average rate of 85.8 per 100,000 movements and lowest in January with an average rate of 19.84 per 100,000 aircraft movements. Similar to wildlife strike counts, variation in the wildlife strike rate was more dramatic within airports than across airports (SD^Overall^ = 56.1; SD^Between^ = 31.3; SD^Within^ = 46.8).

Investigating all reported wildlife strikes together gives a sense of the overall frequency at which wildlife strikes occur. Subsetting wildlife strikes to only those that are considered to be disruptive—i.e., those wildlife strikes that report repair costs, non-repair (other) costs, damage, a negative effect on the flight, aircraft downtime, or any combination of these—however, gives a sense of wildlife strike severity. Together, frequency and severity provide a more complete characterization of wildlife strike risk^8^. The average airport in our sample experienced 0.74 disruptive wildlife strikes per month (Table S1), with the disruptive wildlife strike rate peaking first in April—0.91 per 100,000 movements—and then in October—1.14 per 100,000 movements—which corresponds with Spring and Fall avian migration (SD^Overall^ = 6.0; SD^Between^ = 2.5; SD^Within^ = 5.5).

### Data analyses

To begin our investigation into the relationship between air traffic volume and wildlife strike frequency vis-à-vis the COVID-19 pandemic, we compared air traffic volume, wildlife strikes, and the wildlife strike rate in the pre-pandemic period—March 2019 to December 2019—to those observed during the COVID-19 pandemic—March 2020 to December 2020. These preliminary analyses, which rely on potentially problematic year-over-year comparisons, provided initial evidence that pandemic-induced reductions in air traffic volume were coincident with increases in wildlife strike frequency as measured through the wildlife strike rate.

To estimate the relationship between pandemic-induced reductions in air traffic volume and wildlife strike rates, while also controlling for pre-existing trends in wildlife strike rates and holding constant location-specific confounding factors, we employed fixed-effects negative binomial (NB) regression^25^. NB regression models have been used extensively to model the frequency of traffic accidents^30^ since these data often suffer from overdispersion, as is the case in our data (Table S1). The inclusion of unit-level fixed effects—e.g., airport indicator variables—in non-linear regression models is typically avoided due to biased or inconsistent estimation vis-a-vis the incidental parameter problem^31^. The relatively large time dimension of our panel—e.g., 84 observations per airport—however, supplies confidence that the inclusion of airport fixed-effects is permitted^25,31^. Estimation of the fixed-effects NB regression models are achieved via maximum likelihood in R (version 4.0.4)^32^ using the package ‘fixest’^33^, with model standard errors clustered at the airport-level, which are robust to intragroup correlation in the model disturbances and heteroskedasticity. For each set of estimates presented in our study, we specified and fit a suite of plausible fixed-effects NB regression models. These models include various combinations of control variables including airport fixed effects, month or region-by-month fixed effects, and general or airport-specific year trends. The region classification employed in our analysis were the U.S. Fish and Wildlife Service (USFWS) regions—including Midwest, Mountain Prairie, Northeast, Pacific, Pacific Southwest, Southeast, and Southwest. Like the North American flyways, the USFWS regions also segment the contiguous United States from east to west. However, they offer an additional delineation, distinguishing between the northern and southern territories. In each instance, we selected the most parsimonious model through a comparison of BIC values (Tables S2, S5). An exposure term, which was the log of aircraft movements, was included in all instances to allow for the modeling of wildlife strike *rates* as opposed to *counts.* The model selection results and the full output of our selected models are presented in the Supplementary Tables S2–S7.

In addition to the controls described above, our first set of models included indicator variables for each COVID-19 month of 2020. The inclusion of these indicators allowed us to estimate month-specific deviations in the overall and disruptive wildlife strike rate during March 2020–December 2020 relative to the pre-COVID reference period, on average, all else equal (Fig. 3, Tables S2–S4). Then, to empirically link pandemic-induced reductions in air traffic volume to observed changes in the wildlife strike rate, we estimated an additional set of models. The measure of interest included in these models was the estimated percent reduction (EPR) in aircraft movements over the COVID-19 months of 2020. The derivation of this measure is detailed in the Supplementary Information, Figs. S1 and S2. We interact the EPR with the previously described indicator variables for each COVID-19 month of 2020, which allows us to estimate month-specific relationships between reductions in air traffic volume and wildlife strike rates during March 2020–December 2020, on average, all else equal (Fig. 4, Tables S6, S7). We also tested for a non-linear relationship between reductions in air traffic volume and deviations in the wildlife strike rate through the inclusion of a quadratic term for the EPR (Table S5). For all models, we visually present the estimated parameters of interest as $\text{[IRR}-\text{ 1] }\times \text{ 100}$, which is a transformation of the estimated incident rate ratio (IRR) that provides a more direct interpretation of our results. Full model outputs and the non-transformed IRRs are reported in the Supplementary Information, Tables S3, S4, S6 and S7.

## Supplementary Information

Supplementary Information.

## Data Availability

All data used in our analyses are publicly available. Both air traffic volume and wildlife strike data are provided by the Federal Aviation Administration at https://aspm.faa.gov/opsnet/sys/airport.asp and https://wildlife.faa.gov/home, respectively. The cleaned data and code necessary to replicate the empirical objects of the manuscript can be provided upon request or accessed directly at https://github.com/levialtringer/covid_and_wildlife_strikes.
