# Supplementary material for: The impact of the COVID-19 pandemic on wildlife–aircraft collisions at US airports
Source: Sci Rep. 2023 Jul 18;13:11602. doi: 10.1038/s41598-023-38451-9 (PMC10354106; doi:10.1038/s41598-023-38451-9)
Supplement: Supplementary file 1 — Supplementary Information. [file 41598_2023_38451_MOESM1_ESM.pdf]

Supplementary Information

Manuscript Title:

The Impact of the COVID-19 Pandemic on Wildlife-Aircraft Collisions at US Airports

Authors:

Levi Altringer[ab,1], Sophie C. Mckee[ab], Jason D. Kougher[d], Michael J. Begier[c], and Stephanie A. Shwiff[a]

Affiliations:

[a]United States Department of Agriculture, Animal and Plant Health Inspection Service, Wildlife Services, National Wildlife Research Center, 4101 LaPorte Avenue, Fort Collins, CO 80521, USA

[b]Department of Economics, Colorado State University, Fort Collins, CO, 80523, USA

[c]United States Department of Agriculture, Animal and Plant Health Inspection Service, Wildlife Services, Airport Wildlife Hazards Program, WA, DC, 20250, USA

[d]United States Department of Agriculture, Animal and Plant Health Inspection Service, Wildlife Services, Airport Wildlife Hazards Program, 6100 Columbus Avenue, Sandusky, OH 44870, USA

[1] Corresponding author. *Email:* Levi.Altringer@usda.gov. *Address:* National Wildlife Research Center, 4101 LaPorte Avenue, Fort Collins, CO 80521, USA.

Table of Contents

Supplementary Table S1. Mean monthly aircraft movements, wildlife strikes, and wildlife strike rates in the pre-pandemic (January 2014–December 2019) period……………..……………….…………………….…………….……………….….3

Supplementary Table S2. Model selection exercise for models estimating changes in the (a) overall and (b) disruptive wildlife strike rate during the COVID-19 months of 2020………………….……………………………….……...…………….……4

Supplementary Table S3. Top model estimating changes in the overall wildlife strike rate during the COVID-19 months of 2020……………………….………….…………5

Supplementary Table S4. Top model estimating changes in the disruptive wildlife strike rate during the COVID-19 months of 2020……………………….…………..………6

Supplementary Table S5. Model selection exercise for models estimating the relationship between aircraft movement reductions and changes in the (a) overall and (b) disruptive wildlife strike rate during the COVID-19 months of 2020……….7

Supplementary Table S6. Top model for estimating the relationship between aircraft movement reductions and changes in the overall wildlife strike rate during the COVID-19 months of 2020…………….……..…………………..………….………8

Supplementary Table S7. Top model for estimating the relationship between aircraft movement reductions and changes in the disruptive wildlife strike rate during the COVID-19 months of 2020…….………………..…….…………………….……9

Supplementary Figure S1. Observed and model predicted aircraft movements at three sample airports with heterogeneous experiences over the COVID-19 months of 2020…………………………..…………………………………..…………….….10

Supplementary Figure S2. Estimated percent change in aircraft movements (relative to model expectation) across sample airports during the COVID-19 months of 2020…………………………………………………………………...………11

Supplementary Figure S3. Month-specific model estimated changes in size-specific wildlife strike rates during the COVID-19 months of 2020……………….12

Supplementary Figure S4. Estimating changes in the (a) overall and (b) disruptive wildlife strike rate during the COVID-19 months of 2020 under alternative "treatment" assumptions……..……………..…………..………………………..……….13

Supplementary Figure S5. Estimating the relationship between aircraft movement reductions and changes in the (a) overall and (b) disruptive wildlife strike rate during the COVID-19 months of 2020 under alternative "treatment" assumptions…………………………………………………………………………14

Supplementary Table S1. Mean monthly aircraft movements, wildlife strikes, and wildlife strike rates in the pre-COVID (January 2014–December 2019) period.

	Aircraft Movements	All Strikes		Disruptive Strikes	
		Count	Rate per 100,000*	Count	Rate per 100,000*
All months	26,823.45 (15,849.41)	11.45 (12.04)	51.22 (56.11)	0.74 (1.03)	3.54 (6.00)
January	25,354.60 (14,873.88)	4.03 (4.28)	19.84 (25.45)	0.41 (0.79)	2.19 (5.73)
February	23,458.33 (13,599.18)	4.08 (3.56)	20.73 (19.33)	0.39 (0.72)	2.07 (4.35)
March	27,416.09 (15,925.31)	6.28 (4.83)	27.79 (23.84)	0.60 (0.78)	2.70 (4.39)
April	26,714.53 (15,424.79)	10.32 (8.00)	45.06 (31.94)	0.91 (1.12)	4.02 (5.26)
May	27,650.36 (16,245.19)	14.89 (11.81)	64.46 (50.01)	0.81 (0.98)	3.72 (4.95)
June	27,665.15 (16,479.85)	12.23 (14.84)	52.93 (53.11)	0.55 (0.90)	2.62 (4.83)
July	28,398.16 (17,168.84)	17.60 (16.98)	76.96 (72.22)	0.68 (0.90)	3.24 (5.35)
August	28,354.07 (17,200.73)	18.38 (13.89)	85.80 (84.50)	0.93 (1.20)	4.26 (6.64)
September	26,257.04 (15,713.85)	17.73 (13.20)	83.68 (77.25)	0.96 (1.06)	4.40 (5.14)
October	27,606.40 (16,251.79)	17.08 (13.48)	71.04 (53.76)	1.14 (1.32)	5.16 (6.88)
November	26,153.64 (15,064.94)	9.56 (7.55)	42.22 (31.35)	1.01 (1.16)	5.19 (8.19)
December	26,853.06 (15,502.88)	5.21 (4.67)	24.10 (30.73)	0.52 (0.95)	2.85 (7.79)

Notes: Standard deviations in parentheses. Mean monthly air traffic volume, wildlife strikes, and wildlife strike rates in the pre-pandemic period are calculated from 3,600 airport-month-year observations over the January 2014–December 2019 period.
*The wildlife strike rate is calculated as the count of wildlife strikes divided by the total number of aircraft movements, multiplied by 100,000.

Supplementary Table S2. Model selection exercise for models estimating changes in the (a) overall and (b) disruptive wildlife strike rate during the COVID-19 months of 2020.

Model	Formula	k	BIC	ΔBIC	LogLik
(a) Model selection for the overall strike rate					
5*	strike ~ as.factor(covid_month) + offset(log(total_civil_movements)) + year | airport + month^usfws_region	144	24,757.91	0.00	-11,778.27
6	strike ~ as.factor(covid_month) + offset(log(total_civil_movements)) + airport:year | airport + month^usfws_region	193	24,963.79	205.87	-11,676.81
4	strike ~ as.factor(covid_month) + offset(log(total_civil_movements)) + year | airport + month	72	25,035.76	277.85	-12,217.54
3	strike ~ as.factor(covid_month) + offset(log(total_civil_movements)) | airport + month	71	25,036.03	278.12	-12,221.84
2	strike ~ as.factor(covid_month) + offset(log(total_civil_movements)) | month	22	27,600.77	2,842.86	-13,708.62
1	strike ~ as.factor(covid_month) + offset(log(total_civil_movements))	11	28,823.59	4,065.68	-14,365.91
(b) Model selection for the disruptive strike rate					
3**	disruptive ~ as.factor(covid_month) + offset(log(total_civil_moveme nts)) | airport + month	71	9,530.34	0.00	-4,469.00
4	disruptive ~ as.factor(covid_month) + offset(log(total_civil_moveme nts)) + year | airport + month	72	9,538.65	8.31	-4,468.98
5	disruptive ~ as.factor(covid_month) + offset(log(total_civil_moveme nts)) + year | airport + month^usfws_region	144	9,891.87	361.54	-4,345.25
2	disruptive ~ as.factor(covid_month) + offset(log(total_civil_moveme nts)) | month	22	9,940.14	409.81	-4,878.30
1	disruptive ~ as.factor(covid_month) + offset(log(total_civil_moveme nts))	11	10,034.63	504.30	-4,971.43
6	disruptive ~ as.factor(covid_month) + offset(log(total_civil_moveme nts)) + airport:year | airport + month^usfws_region	193	10,247.40	717.07	-4,318.62

Notes: Models were estimated via fenegbin() from the 'fixest' package in R (version 4.0.4).
*Coefficient estimates presented in Table S3.
**Coefficient estimates presented in Table S4.

Supplementary Table S3. Top model estimating changes in the overall wildlife strike rate during the COVID-19 months of 2020.

ML estimation, family = Negative Binomial, Dep. Var.: strike
Observations: 4,200
Offset: log(total_civil_movements)
Fixed-effects: airport: 50, month^usfws_region: 84
Standard-errors: Clustered (airport)

	Estimate	Std. Error	t-value	Pr(>|t|)	IRR	IRR 90% C.I.
March 2020	0.041	0.086	0.480	0.631	1.042	[0.894, 1.191]
April 2020	0.093	0.103	0.909	0.363	1.098	[0.912, 1.284]
May 2020	0.192**	0.081	2.379	0.017	1.212	[1.051, 1.374]
June 2020	0.320***	0.086	3.720	0.000	1.377	[1.182, 1.571]
July 2020	0.176**	0.076	2.315	0.021	1.192	[1.043, 1.341]
August 2020	0.122*	0.069	1.770	0.077	1.129	[1.002, 1.257]
September 2020	0.109**	0.055	1.994	0.046	1.116	[1.015, 1.216]
October 2020	-0.035	0.066	-0.532	0.595	0.965	[0.860, 1.071]
November 2020	-0.060	0.076	-0.790	0.430	0.942	[0.824, 1.059]
December 2020	-0.037	0.092	-0.404	0.686	0.963	[0.818, 1.109]
Time trend (Year)	0.017*	0.009	1.785	0.074	1.017	[1.001, 1.032]
Over-dispersion Parameter: Theta	7.048	0.599	11.762	0.000		

Log-Likelihood: -11,778.3
BIC: 24,757.9
Adj. Pseudo R2: 0.171745
Squared Cor.: 0.720443

Notes: Coefficient estimates that satisfy traditional levels of statistical significance indicated by * $p < 0.1$, ** $p < 0.05$, *** $p < 0.01$. The IRR is calculated as *exp*(Estimate). The IRR 90% C.I. are calculated as IRR ± 1.645**SE*(IRR) where the *SE*(IRR)—the transformed standard errors—are achieved via the delta method.

Supplementary Table S4. Top model estimating changes in the disruptive wildlife strike rate during the COVID-19 months of 2020.

ML estimation, family = Negative Binomial, Dep. Var.: disruptive
Observations: 4,200
Offset: log(total_civil_movements)
Fixed-effects: airport: 50, month: 12
Standard-errors: Clustered (airport)

	Estimate	Std. Error	t-value	Pr(>|t|)	IRR	IRR 90% C.I.
March 2020	0.126	0.215	0.585	0.559	1.134	[0.733, 1.535]
April 2020	0.133	0.293	0.456	0.649	1.143	[0.592, 1.694]
May 2020	0.103	0.240	0.428	0.668	1.108	[0.671, 1.545]
June 2020	0.545**	0.223	2.446	0.014	1.725	[1.092, 2.359]
July 2020	0.251	0.182	1.377	0.169	1.286	[0.900, 1.671]
August 2020	0.100	0.166	0.604	0.546	1.105	[0.803, 1.407]
September 2020	0.138	0.159	0.867	0.386	1.148	[0.847, 1.449]
October 2020	0.117	0.161	0.722	0.470	1.124	[0.825, 1.422]
November 2020	-0.182	0.205	-0.884	0.377	0.834	[0.552, 1.116]
December 2020	-0.384	0.317	-1.212	0.226	0.681	[0.326, 1.036]
Over-dispersion Parameter: Theta	4.829	1.235	3.911	0.000		

Log-Likelihood: -4,469.0
BIC: 9,530.3
Adj. Pseudo R2: 0.062813
Squared Cor.: 0.165404

Notes: Coefficient estimates that satisfy traditional levels of statistical significance indicated by * $p < 0.1$, ** $p < 0.05$, *** $p < 0.01$. The IRR is calculated as *exp*(Estimate). The IRR 90% C.I. are calculated as IRR ± 1.645**SE*(IRR) where the *SE*(IRR)—the transformed standard errors—are achieved via the delta method.

Supplementary Table S5. Model selection exercise for models estimating the relationship between aircraft movement reductions and changes in the (a) overall and (b) disruptive wildlife strike rate during the COVID-19 months of 2020.

Model	Formula	k	BIC	∆BIC	LogLik
(a) Model selection for the overall strike rate					
5*	strike ~ epr:as.factor(covid_month) + offset(log(total_civil_movements)) + year | airport + month^usfws_region	145	24,762.60	0.00	-11,776.45
6	strike ~ epr:as.factor(covid_month) + epr2:as.factor(covid_month) + offset(log(total_civil_movements)) + year | airport + month^usfws_region	156	24,842.66	80.05	-11,770.59
7	strike ~ epr:as.factor(covid_month) + offset(log(total_civil_movements)) + airport:year | airport + month^usfws_region	194	24,970.65	208.04	-11,676.07
3	strike ~ epr:as.factor(covid_month) + offset(log(total_civil_movements)) | airport + month	72	25,041.55	278.94	-12,220.43
4	strike ~ epr:as.factor(covid_month) + offset(log(total_civil_movements)) + year | airport + month	73	25,043.54	280.94	-12,217.26
8	strike ~ epr:as.factor(covid_month) + epr2:as.factor(covid_month) + offset(log(total_civil_movements)) + airport:year | airport + month^usfws_region	205	25,048.20	285.59	-11,668.96
2	strike ~ epr:as.factor(covid_month) + offset(log(total_civil_movements)) | month	23	27,620.44	2,857.84	-13,714.28
1	strike ~ epr:as.factor(covid_month) + offset(log(total_civil_movements))	12	28,864.42	4,101.82	-14,382.15
(b) Model selection for the disruptive strike rate					
3**	disruptive ~ epr:as.factor(covid_month) + offset(log(total_civil_movements)) | airport + month	72	9,537.84	0.00	-4,468.58
4	disruptive ~ epr:as.factor(covid_month) + offset(log(total_civil_movements)) + year | airport + month	73	9,546.16	8.32	-4,468.57
5	disruptive ~ epr:as.factor(covid_month) + offset(log(total_civil_movements)) + year | airport + month^usfws_region	145	9,897.76	359.91	-4,344.02
2	disruptive ~ epr:as.factor(covid_month) + offset(log(total_civil_movements)) | month	23	9,950.82	412.98	-4,879.47
6	disruptive ~ epr:as.factor(covid_month) + epr2:as.factor(covid_month) + offset(log(total_civil_movements)) + year | airport + month^usfws_region	156	9,978.15	440.30	-4,338.33
1	disruptive ~ epr:as.factor(covid_month) + offset(log(total_civil_movements))	12	10,045.82	507.98	-4,972.85
7	disruptive ~ epr:as.factor(covid_month) + offset(log(total_civil_movements)) + airport:year | airport + month^usfws_region	194	10,253.90	716.06	-4,317.69
8	disruptive ~ epr:as.factor(covid_month) + epr2:as.factor(covid_month) + offset(log(total_civil_movements)) + airport:year | airport + month^usfws_region	205	10,334.22	796.37	-4,311.97

Notes: Models were estimated via fenegbin() from the ‘fixest’ package in R (version 4.0.4).
*Coefficient estimates presented in Table S6.
**Coefficient estimates presented in Table S7.

Supplementary Table S6. Top model for estimating the relationship between aircraft movement reductions and changes in the overall wildlife strike rate during the COVID-19 months of 2020.

ML estimation, family = Negative Binomial, Dep. Var.: strike
Observations: 4,200
Offset: log(total_civil_movements)
Fixed-effects: airport: 50, month^usfws_region: 84
Standard-errors: Clustered (airport)

	Estimate	Std. Error	t-value	Pr(>|t|)	IRR	IRR 90% C.I.
EPR × Pre-COVID	0.0010	0.0038	0.2712	0.7862	1.001	[0.9947, 1.0074]
EPR × March 2020	0.0038	0.0050	0.7640	0.4449	1.0038	[0.9956, 1.0121]
EPR × April 2020	0.0016	0.0015	1.0772	0.2814	1.0016	[0.9991, 1.0042]
EPR × May 2020	0.0025**	0.0012	2.0644	0.0390	1.0025	[1.0005, 1.0046]
EPR × June 2020	0.0061***	0.0015	4.2066	0.0000	1.0061	[1.0037, 1.0085]
EPR × July 2020	0.0045**	0.0016	2.8574	0.0043	1.0045	[1.0019, 1.0071]
EPR × August 2020	0.0034**	0.0015	2.2898	0.0220	1.0034	[1.0010, 1.0059]
EPR × September 2020	0.0030**	0.0014	2.1886	0.0286	1.003	[1.0007, 1.0053]
EPR × October 2020	0.0011	0.0016	0.6465	0.5180	1.0011	[0.9984, 1.0038]
EPR × November 2020	-0.0005	0.0019	-0.2677	0.7889	0.9995	[0.9964, 1.0026]
EPR × December 2020	0.0007	0.0027	0.2616	0.7936	1.0007	[0.9963, 1.0051]
Time trend (Year)	0.0144	0.0088	1.6300	0.1031	1.0145	[0.9998, 1.0292]
Over-dispersion Parameter: Theta	7.0627	0.6085	11.6072	0.0000		

Log-Likelihood: -11,776.4
BIC: 24,762.6
Adj. Pseudo R2: 0.171802
Squared Cor.: 0.720703

Notes: Coefficient estimates that satisfy traditional levels of statistical significance indicated by * $p < 0.1$, ** $p < 0.05$, *** $p < 0.01$. The IRR is calculated as *exp*(Estimate). The IRR 90% C.I. are calculated as IRR ± 1.645**SE*(IRR) where the *SE*(IRR)—the transformed standard errors—are achieved via the delta method.

Supplementary Table S7. Top model for estimating the relationship between aircraft movement reductions and changes in the disruptive wildlife strike rate during the COVID-19 months of 2020.

ML estimation, family = Negative Binomial, Dep. Var.: disruptive
Observations: 4,200
Offset: log(total_civil_movements)
Fixed-effects: airport: 50, month: 12
Standard-errors: Clustered (airport)

	Estimate	Std. Error	t-value	Pr(>|t|)	IRR	IRR 90% C.I.
EPR × Pre-COVID	0.0062	0.0066	0.9283	0.3532	1.0062	[0.9952, 1.0171]
EPR × March 2020	0.0083	0.0111	0.7414	0.4585	1.0083	[0.9898, 1.0268]
EPR × April 2020	0.0011	0.0046	0.2436	0.8075	1.0011	[0.9935, 1.0088]
EPR × May 2020	0.0026	0.0036	0.7348	0.4625	1.0026	[0.9968, 1.0085]
EPR × June 2020	0.0110***	0.0038	2.8947	0.0038	1.0111	[1.0047, 1.0174]
EPR × July 2020	0.0039	0.0042	0.9402	0.3471	1.0039	[0.9970, 1.0109]
EPR × August 2020	0.0018	0.0040	0.4549	0.6492	1.0018	[0.9952, 1.0085]
EPR × September 2020	0.0021	0.0038	0.5539	0.5796	1.0021	[0.9958, 1.0084]
EPR × October 2020	0.0061	0.0038	1.6201	0.1052	1.0062	[0.9999, 1.0124]
EPR × November 2020	-0.0004	0.0057	-0.0672	0.9464	0.9996	[0.9903, 1.0089]
EPR × December 2020	-0.0043	0.0095	-0.4528	0.6507	0.9957	[0.9801, 1.0113]
Over-dispersion Parameter: Theta	4.8111	1.2295	3.9129	0.0001		

Log-Likelihood: -4,468.6
BIC: 9,537.8
Adj. Pseudo R2: 0.062693
Squared Cor.: 0.164593

Notes: Coefficient estimates that satisfy traditional levels of statistical significance indicated by * $p < 0.1$, ** $p < 0.05$, *** $p < 0.01$. The IRR is calculated as *exp*(Estimate). The IRR 90% C.I. are calculated as IRR ± 1.645**SE*(IRR) where the *SE*(IRR)—the transformed standard errors—are achieved via the delta method.

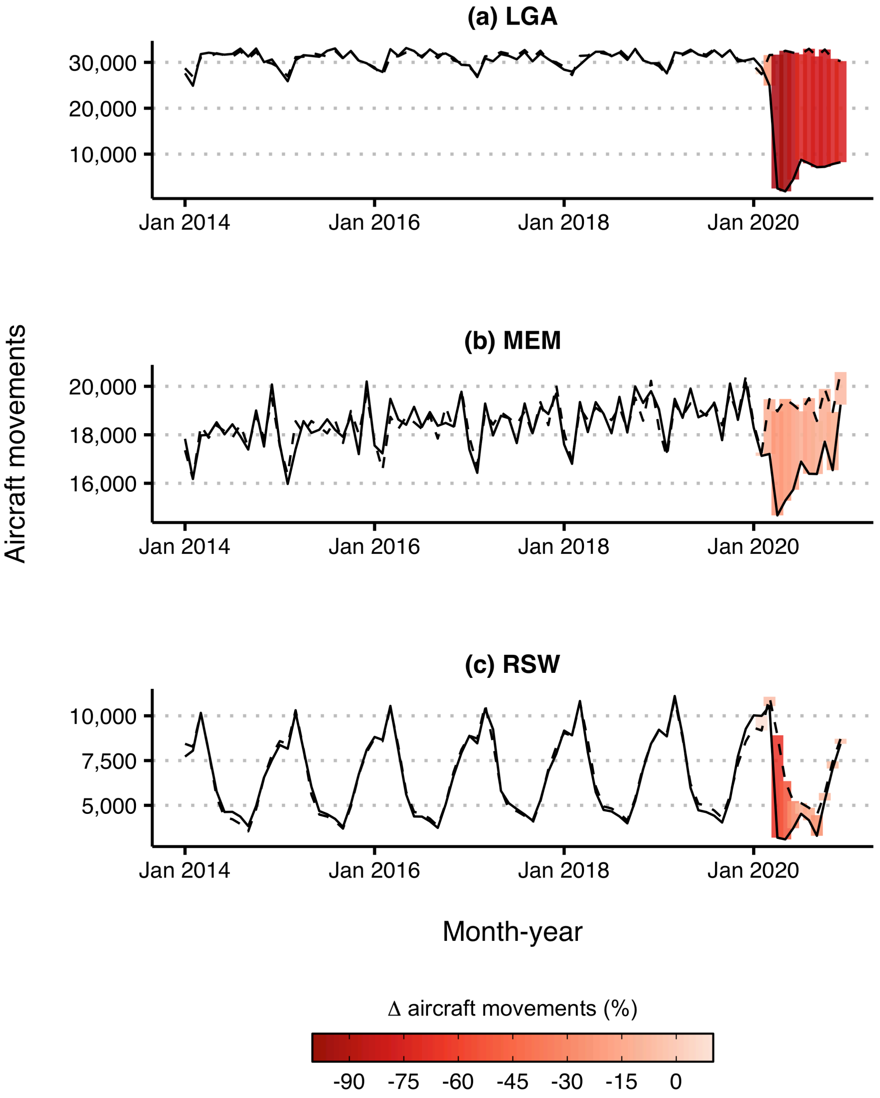

Supplementary Figure S1. Observed (solid line) and model predicted (dashed line) aircraft movements at three sample airports with heterogeneous experiences over the COVID-19 months of 2020— (a) LaGuardia Airport (LGA), (b) Memphis International Airport (MEM), (c) Southwest Florida International Airport (RSW). Model predicted aircraft movements are obtained via a simple linear regression model, $AM_{it} = \alpha + \boldsymbol{\eta} A_i + \boldsymbol{\gamma} M_{it} + \boldsymbol{\delta} t_i + \varepsilon_{it}$, where aircraft movements (AM) at airport *i* and time (month-year) *t* are specified to be a linear function of airport fixed-effects ($\boldsymbol{\eta} A_i$), airport-specific month effects ($\boldsymbol{\gamma} M_{it}$), and airport-specific linear time trends ($\boldsymbol{\delta} t_i$). This model is estimated on data from the pre-COVID-19 period—i.e., January 2014 to February 2020—and used to predict monthly aircraft operations for each airport over the entire sample period, including March 2020 to December 2020. The predictive performance of this simple model on pre-COVID-19 data is strong ($R^2 = 0.997$), providing a reliable counterfactual for aircraft movements at each airport had the COVID-19 pandemic not occurred. All sample airports experienced decreases in aircraft movements (relative to model expectation) during the COVID-19 months of 2020 (see Fig. S2). As the figure above indicates, however, some experienced larger, long-lasting reductions in air traffic volume while others experienced more mild reductions and/or recovered more quickly.

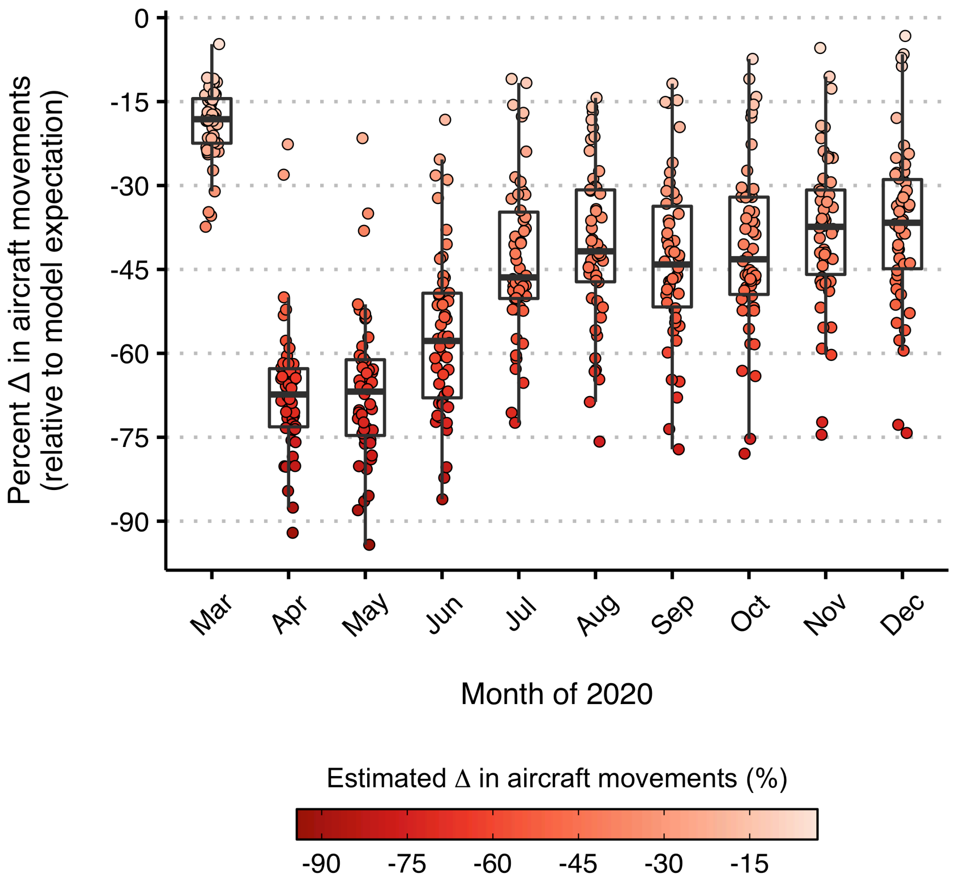

Supplementary Figure S2. Estimated percent change in aircraft movements (relative to model expectation) across sample airports during the COVID-19 months of 2020. The points, which are jittered for readability, are calculated as airport-by-month differences between predicted and observed aircraft movements—i.e., the dashed and solid lines shown in Figure S1. The overlaying boxplots present the 25th percentile (bottom of each box), median (bold line within each box), and the 75th percentile (top of each box) of changes in aircraft movements during each month of the COVID-19 pandemic in 2020. It is the measure plotted in this figure, multiplied by -1, that constitutes our continuous measure of "treatment", the estimated percent reduction (EPR) in aircraft movements, in our fixed-effect negative binomial regression models (see Tables S5-S7).

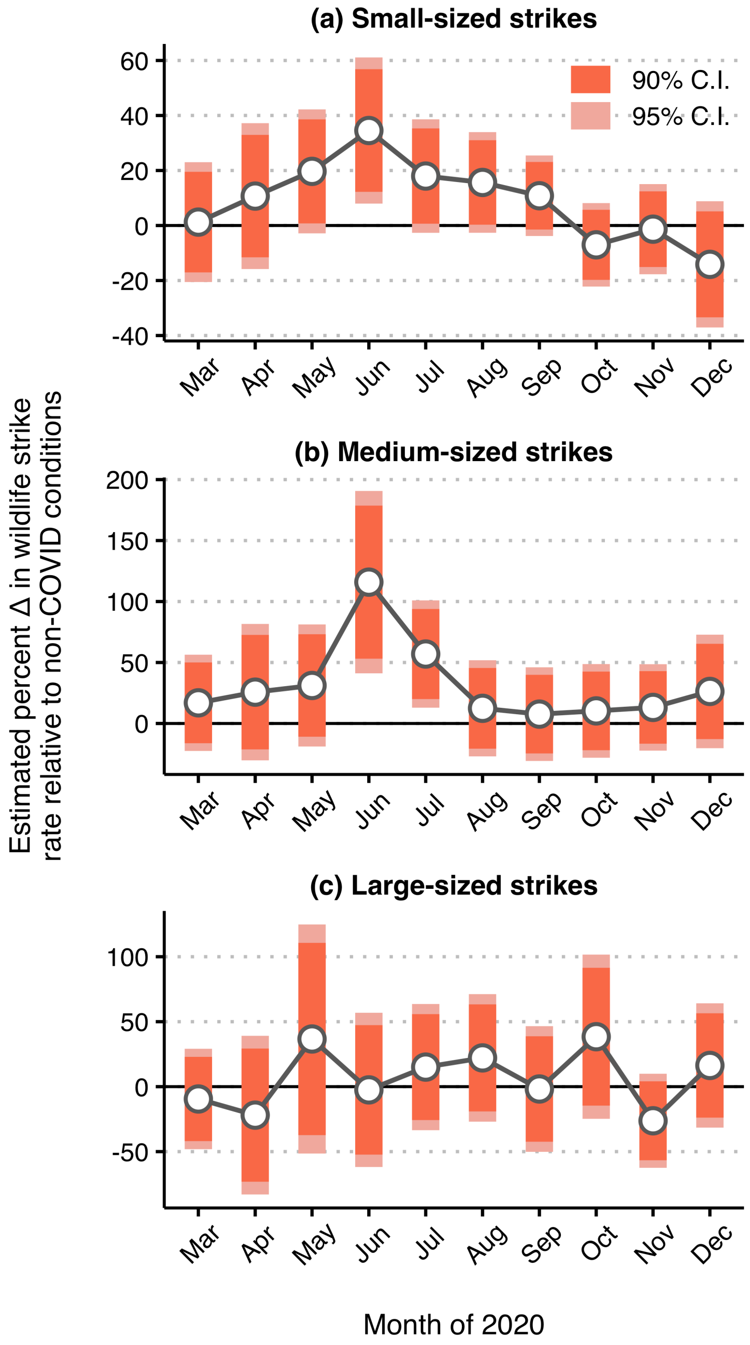

Supplementary Figure S3. Month-specific model estimated changes in size-specific wildlife strike rates during the COVID-19 months of 2020—(a) small (≤ 442g), (b) medium (≥ 443g & ≤ 1500g), and (c) large (> 1500g). The connected points in each panel report the model estimated percent change in the wildlife strike rate during the COVID months of 2020, all else equal. The models used to generate these size-specific strike rate estimates are identical to the preferred model in Supplementary Information Table S2a—i.e., Model 5. The error bars provide the associated 90% and 95% intervals of confidence that surround each estimate.

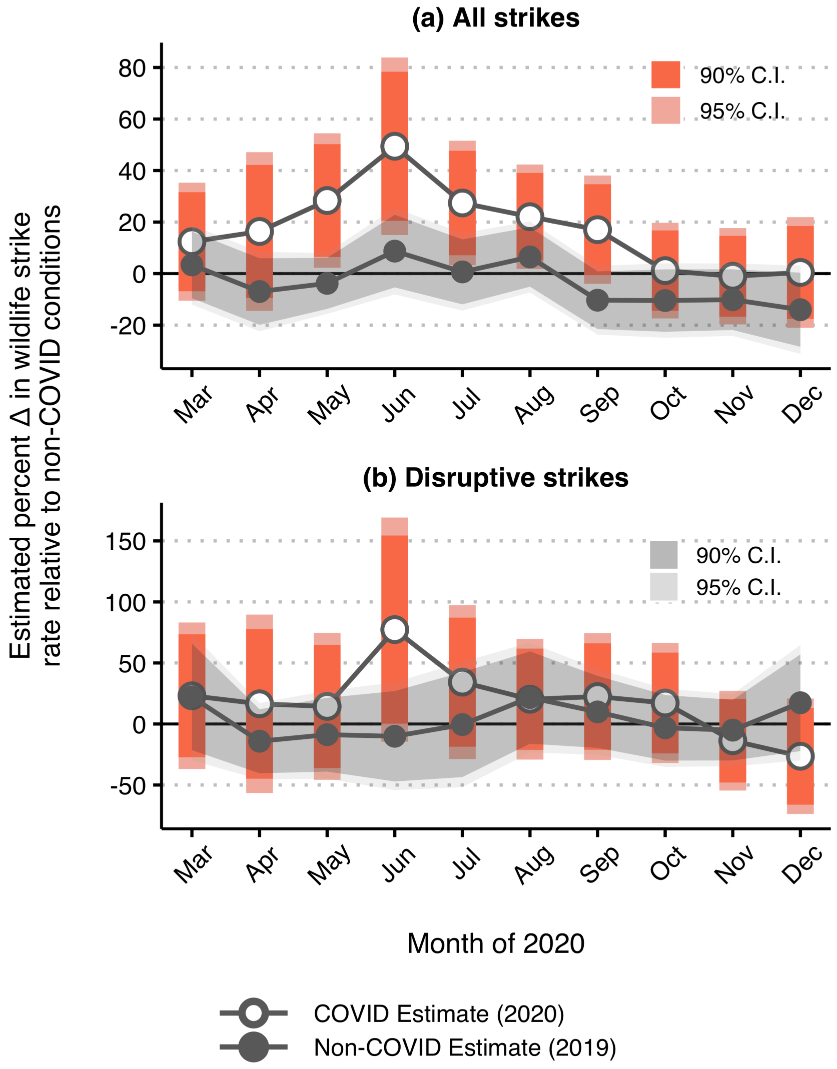

Supplementary Figure S4. Comparing estimated changes in the (a) overall and (b) disruptive wildlife strike rate during the COVID-19 months of 2020 under alternative "treatment" assumptions. Specifically, the preferred models from Supplementary Table S2 are re-estimated under two alternative strategies. First, the sample of analysis is subset to include observations from January 2014-December 2019 and "treatment" is assigned to the COVID-19 months of 2019 (March-December)—we call this the "Non-COVID" strategy. Second, the sample of analysis is subset to include observations from January 2014-December 2018 and January 2020-December 2020—i.e., 2019 data is excluded. "Actual treatment" is maintained for the COVID-19 months of 2020 (March-December) since these were, in fact, the months of data that were affected by the COVID-19 pandemic)—we call this the "COVID" strategy. The parameters of interest, estimated under the "Non-COVID" and "COVID" strategies, are then compared. If the regression models sufficiently predict variation in pre-COVID wildlife strike rates, then the expectation is that the "Non-COVID"—i.e., 2019—estimates should not significantly deviate from 0. Further, if pandemic-induced reductions in air traffic volume did, in fact, result in elevated strike rates that are unanticipated by pre-COVID data, the "COVID" estimates should significantly diverge from 0, in addition to significantly deviating from the "Non-COVID" estimates. The estimates presented in panel (a) validate both expectations during the months May-September while the "COVID" and "Non-COVID" estimates in panel (b) only significantly depart from each other in June.

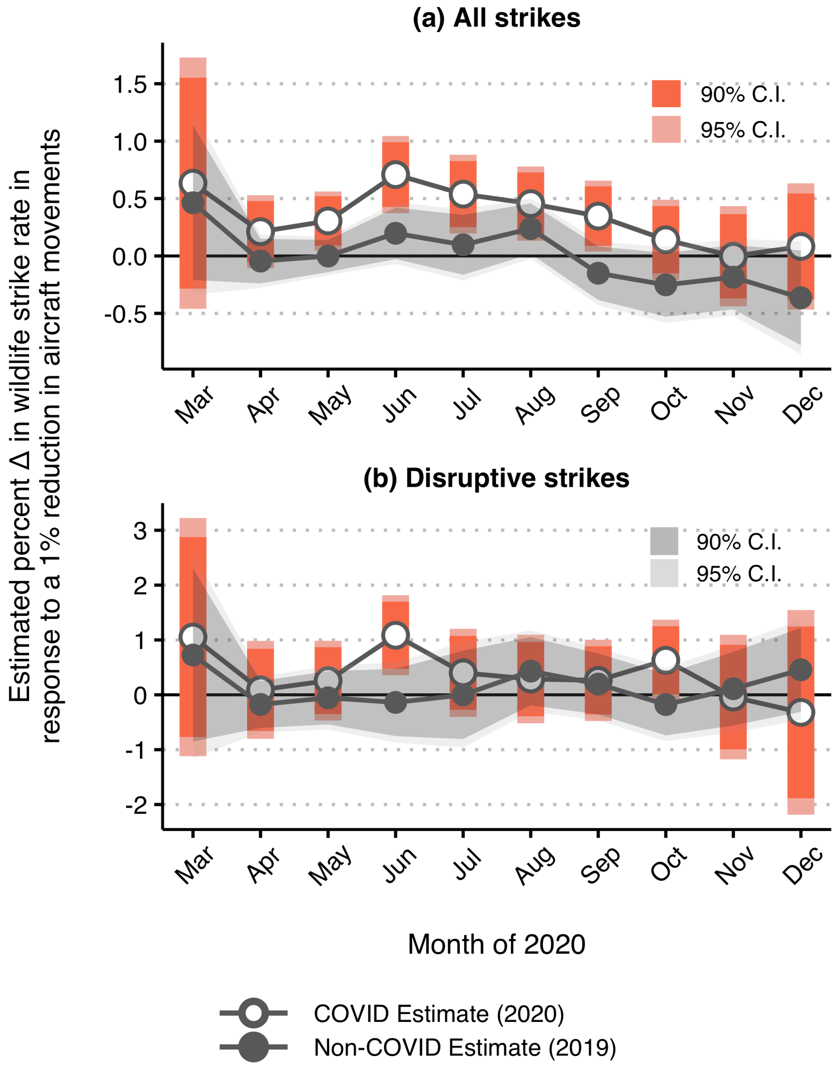

Supplementary Figure S5. Comparing the estimated relationship between aircraft movement reductions and changes in the (a) overall and (b) disruptive wildlife strike rate during the COVID-19 months of 2020 under alternative "treatment" assumptions. Specifically, the preferred models from Supplementary Table S5 are re-estimated under two alternative strategies. First, the sample of analysis is subset to include observations from January 2014-December 2019 and "treatment" is assigned to the COVID-19 months of 2019 (March-December)—i.e., pandemic-induced reductions in air traffic volume (EPR) are assigned to March-December of 2019. We call this the "Non-COVID" strategy. Second, the sample of analysis is subset to include observations from January 2014-December 2018 and January 2020-December 2020—i.e., 2019 data is excluded. "Actual treatment" is maintained for the COVID-19 months of 2020 (March-December) since these were, in fact, the months of data that were affected by the COVID-19 pandemic—we call this the "COVID" strategy. The parameters of interest, estimated under "Non-COVID" and "COVID" strategies, are then compared. If the regression models sufficiently predict variation in pre-COVID wildlife strike rates, then pandemic-induced reductions in air traffic volume during 2020 should not be significantly associated with deviations in wildlife strike rates in 2019. However, if pandemic-induced reductions in air traffic volume did have some influence on wildlife strike rates in 2020, the "COVID" estimates should significantly deviate from 0, in addition to significantly diverging from the "Non-COVID" estimates.
